# The value of subtraction MRI in detection of amyloid-related imaging abnormalities with oedema or effusion in Alzheimer’s patients: An interobserver study

**DOI:** 10.1007/s00330-017-5022-6

**Published:** 2017-09-27

**Authors:** Roland M. Martens, Arianne Bechten, Silvia Ingala, Ronald A. van Schijndel, Vania B. Machado, Marcus C. de Jong, Esther Sanchez, Derk Purcell, Michael H. Arrighi, Robert H. Brashear, Mike P. Wattjes, Frederik Barkhof

**Affiliations:** 1 Department of Radiology and Nuclear Medicine, Neuroscience Campus Amsterdam, VU University Medical Center, PO Box 7057, 1007 MB Amsterdam, The Netherlands; 2 Department of Radiology, California Pacific Medical Center, San Francisco, CA USA; 3 BioClinica Inc, Newark, CA USA; 4 Janssen Alzheimer Immunotherapy Research & Development, LLC, South San Francisco, CA USA; 5 Institutes of Neurology and Healthcare Engineering, University College London, London, UK

**Keywords:** Alzheimer's disease (AD), Amyloid beta (Aβ), Immunotherapy, ARIA (amyloid-related imaging abnormalities), MRI (magnetic resonance imaging)

## Abstract

**Background:**

Immunotherapeutic treatments targeting amyloid-β plaques in Alzheimer’s disease (AD) are associated with the presence of amyloid-related imaging abnormalities with oedema or effusion (ARIA-E), whose detection and classification is crucial to evaluate subjects enrolled in clinical trials.

**Purpose:**

To investigate the applicability of subtraction MRI in the ARIA-E detection using an established ARIA-E-rating scale.

**Methods:**

We included 75 AD patients receiving bapineuzumab treatment, including 29 ARIA-E cases. Five neuroradiologists rated their brain MRI-scans with and without subtraction images. The accuracy of evaluating the presence of ARIA-E, intraclass correlation coefficient (ICC) and specific agreement was calculated.

**Results:**

Subtraction resulted in higher sensitivity (0.966) and lower specificity (0.970) than native images (0.959, 0.991, respectively). Individual rater detection was excellent. ICC scores ranged from excellent to good, except for gyral swelling (moderate). Excellent negative and good positive specific agreement among all ARIA-E imaging features was reported in both groups. Combining sulcal hyperintensity and gyral swelling significantly increased positive agreement for subtraction images.

**Conclusion:**

Subtraction MRI has potential as a visual aid increasing the sensitivity of ARIA-E assessment. However, in order to improve its usefulness isotropic acquisition and enhanced training are required. The ARIA-E rating scale may benefit from combining sulcal hyperintensity and swelling.

***Key Points*:**

• *Subtraction technique can improve detection amyloid-related imaging-abnormalities with edema/effusion in Alzheimer’s patients.*

• *The value of ARIA-E detection, classification and monitoring using subtraction was assessed.*

• *Validation of an established ARIA-E rating scale, recommendations for improvement are reported.*

• *Complementary statistical methods were employed to measure accuracy, inter-rater-reliability and specific agreement.*

## Introduction

Alzheimer’s disease (AD) is a progressive neurodegenerative disease defined by the deposition of amyloid-ß (Aß) plaques and τ-neurofibrillary tangles in the brain, leading to cognitive impairment and neuronal loss [1, 2]. To date, despite multiple investigated treatment approaches, no curative options exist. Aβ is a promising target for immunotherapy, and both active and passive immunisation strategies aiming at removal of Aß-plaques and prevention of neurodegeneration are currently being evaluated in a number of trials [3–6].

Amyloid-related image abnormalities (ARIA) were reported on brain MRI of AD subjects enrolled in immunisation trials and they are likely related to the clearance mechanism of Aβ [7]. Clinically, ARIA cases can be associated with non-specific signs and symptoms and reduction in cognitive performance as assessed by the Mini-Mental-State-Examination (MMSE), though most cases remain asymptomatic [8–13]. Based on their radiological appearance, these abnormalities are subdivided into ARIA-H, representing hemosiderin deposits and microbleeds in the brain parenchyma resulting from blood leakage from adjacent brain vessels, and ARIA-E, showing parenchymal vasogenic oedema and/or sulcal effusion [7]. Considering the variety of pathologies with similar radiological appearances, the risk of misidentification and misinterpretation of ARIA-E abnormalities is significant and may affect patients’ monitoring and eventually the outcome of clinical trials [14].

A visual rating scale allowing an easily applicable characterisation of ARIA-E in all brain regions was developed to estimate the severity of these abnormalities [4]. Bechten et al. recently demonstrated that this rating scale was simple and robust and showed a high agreement both in the identification and determination of ARIA-E severity and in the regional categorisation of the various manifestations [15]. In order to improve the classification of ARIA cases and scoring we explored the applicability of adding subtraction images. The subtraction technique, in which one scan is digitally subtracted from a co-registered second scan, has already proven to be valuable in the detection, quantification and monitoring of lesions over time in the setting of multiple sclerosis and glioblastoma multiforme. In both the latter disorders, the effect of repositioning and enhancing contrast between the active lesions and the non-active background must be taken into account [16–19]. The aim of this study was to assess the value of using registered subtraction images (1) for detection and (2) classification of ARIA-E, and (3) to determine the inter-rater agreement using an established ARIA-E rating scale.

## Methods

### Patient group and study design

We included 75 subjects with AD from a phase II, multicentre, randomised, double-blind, placebo-controlled multiple ascending dose study of bapineuzumab, a humanised monoclonal antibody targeting Aβ [20]. The phase II multicentre study was performed at 30 different sites in the USA between April 2005 and March 2008. 234 patients were randomly assigned to receive intravenous bapineuzumab or a placebo, in a ratio of 8:7, in one of four sequential dose cohorts. Volumetric and safety baseline and follow-up fluid-attenuated inversion recovery (FLAIR) MRI scans were performed prior to first infusion and 6 weeks after treatment, respectively, and then patients were scanned subsequently at intervals of 13 weeks up to week 71 [20].

For the current study we assessed 75 AD patients from the above-described multicentre study, including 29 positive ARIA-E and 46 negative ARIA-E cases. Follow-up scans were obtained at regular intervals. For positive ARIA-E cases we selected the first scan on which the ARIA-E was seen and compared this with the baseline scan. Table 1 shows the baseline subject characteristics.

**Table 1:** Demographics and baseline information of the Alzheimer’s disease (AD) patients included in this study

AD subjects	No.	%
Total	75	100
Female	46	61.3
Male	29	38.7
In initial study ^8^	Mean	SD
Age (y)	67.36	8.35
Baseline MMSE*	20.83	2.92
Baseline DAD**	86.27	14.45
ApoE allele frequency		
0	16	21.92
1	37	50.68
2	20	27.40
Assigned dose in mg/kg		
0.15	13	17.33
0.5	17	22.67
1	23	30.67
2	22	29.33

*MMSE* Mini Mental State Examination, *DAD* Disability Assessment for Dementia

### MRI and subtraction images

**Fig. 1:**
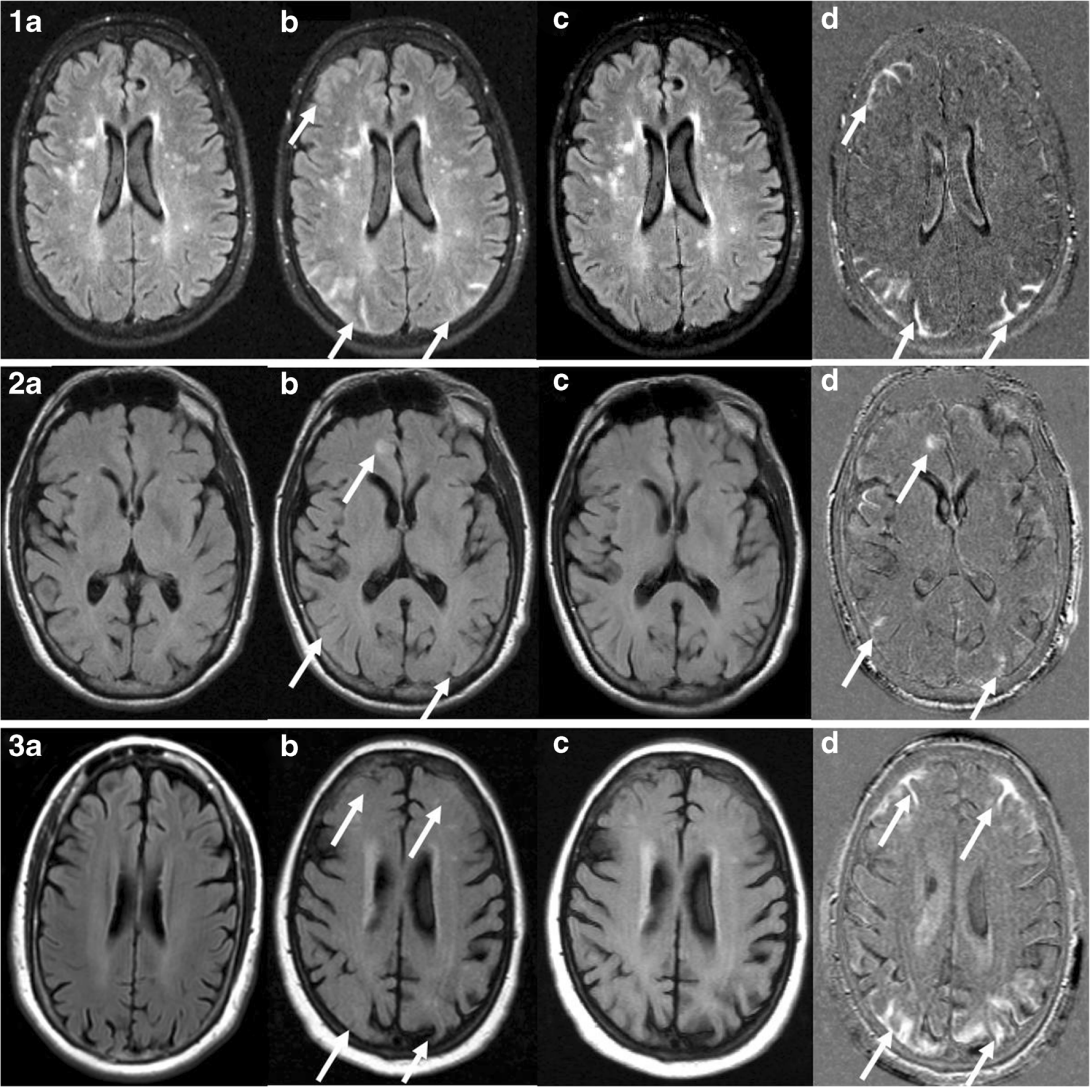
Three different cases of amyloid-related image abnormalities with vasogenic oedema and/or sulcal effusion (ARIA-E). Baseline and follow-up axial FLAIR scans (vertical section A and B, respectively) showing multiple lesions. Section C illustrates the registration image of the follow-up scan to the baseline scan. Section D shows the subtraction image aiding in the detection, distinguishing or exclusion of ARIA-E findings. (**1**) Signal hyperintensities (especially on the right hemisphere) are visible on the follow-up FLAIR axial image. The generated subtraction image helps in differentiating between parenchymal and sulcal hyperintensities. Note subtraction artefacts in the ventricles due to poor CSF suppression. (**2**) Subtle ARIA-E abnormalities are barely visible on FLAIR scan but can be more easily detectable on subtraction images (arrows). (**3**) Gyral swelling is hardly detectable at axial FLAIR but it is clearly distinguishable in the subtraction image (arrows). Note that the slice angulation between baseline and follow-up is quite different, but the registered baseline is nevertheless relative comparable to the follow-up

Each patient underwent a baseline MRI including an axial FLAIR sequence before treatment and follow-up scans at scheduled intervals. At each site, MRIs were performed with identical parameters. However, among sites the scanning protocols differed slightly. Mean echo time (TE) was 129.6 ms (interpatient range 79–159.5 ms); mean repetition time (TR) 9,374.8 ms (range 9,002–11,002 ms); flip angle (90, 150 or 180). The voxel size was 0.51x0.51 mm, 0.88x0.88 mm, 0.90x0.90 mm, 0.94x0.94 mm or 1.02x1.02 mm; slice thickness 5 mm. Axial T2-weighted and FLAIR sequences were anonymised. FLAIR images were used to generate subtraction images. T2-weighted images were not employed because the high signal intensities due to partial volume averaging effects from adjacent blood or CSF could mimic parenchymal lesions and cause artefacts [19–21]. The follow-up images were registered to baseline images through an automatic voxel-based registration algorithm relying on mutual information as the matching criterion [22, 23]. Linear intra- and intermodal brain image registration were obtained through FSL Flirt software program and trilinear interpolation was employed for both image interpolation and reslicing of data [21, 24, 25]. First a global scaling was applied based on the ratio of the average brain signal intensity (based on FSL BET) of the baseline and follow-up images (native images; NAT) [26]. Then the baseline scan (Fig. 1a) was registered to the follow-up scan (Fig. 1b) resulting in a new registered baseline scan (Fig. 1c). The registered baseline scan was subtracted from the follow-up scan accordingly. This resulted in the pixel-enhanced subtraction image (subtraction images; SUB) (Fig. 1d), highlighting changes in time.

### Image analysis

Five experienced neuroradiologists independently reviewed the scans of the 75 subjects included. Reading results of the scans of the phase II bapineuzumab study were used as gold standard for ARIA-E cases, which had been performed previously by two neuroradiologists independently (kappa=0.76) followed by consensus reached over all FLAIR MRIs from the 262 patients [7]. The raters were blinded to clinical information and unaware of the gold standard ARIA-E rating scores. Prior to scoring, the neuroradiologists were provided a web-based introduction regarding ARIA-E and a training set on how to use the rating scale. The scans were presented in random order to the nheuroradiologists on a web platform, which allowed the raters to compare the NAT and SUB, to perform measurements and to score each case. Each rater was requested to identify ARIA-E using baseline and follow-up axial FLAIR MR images without the use of the SUB. Twelve months later, the scans of the same subjects were presented in a new random order to the same raters, who re-evaluated them with the use of the subtraction MRI as an additional tool.

### ARIA-E rating scale

Table 2 reports the rating scale for ARIA-E [4]. For hyperintensities or gyral swelling, ratings are performed according to the anatomical location in terms of lobe and side (L/R), resulting in scores for six regions bilaterally: frontal, parietal, temporal and occipital lobes, central region (including basal ganglia, thalamus, internal and external capsules, corpus callosum and insula) and infratentorial region (brainstem and cerebellum). Within each region, the score depends on the spatial extent and multifocality of the abnormality. In the case of abnormalities involving multiple locations, their maximum in-plane diameter in each lobe is measured and scored. The regional scores on each side of the brain (L/R) are summed up for each ARIA-E subtype and the highest score of the 3 ARIA-E imaging features subtypes contributed to the score of the region.

**Table 2:** Amyloid-related image abnormalities with vasogenic oedema and/or sulcal effusion (ARIA-E) rating scale

FLAIR finding	Baseline scoring	ARIA-E after treatment initiation	Evolution of ARIA-E
Parenchymal hyperintensity	ARWMC score by 1. Lesion size 2. Region and side (if yes – ARIA-E like?)	Count new focal lesions 1. By region and side 2. By largest cross-sectional diameter Score 0- 0 Score 1- Monofocal ≤ 2 cm Score 2- Multifocal ≤ 2 cm Score 3- Any lesion > 2 but < 4 cm Score 4- Any lesion >4 cm Score 5- Entire lobe	Increase ARIA-E ARIA-E unchanged Partial resolution Full resolution N/A initial identification N/A other pathology
Sulcal hyperintensity	Yes / No	Count new focal lesions 1. By region and side 2. By largest cross-sectional diameter Score 0- 0 Score 1- Monofocal ≤ 2 cm Score 2- Multifocal ≤ 2 cm Score 3- Any lesion > 2 but < 4 cm Score 4- Any lesion >4 cm Score 5- Entire lobe	Increase ARIA-E ARIA-E unchanged Partial resolution Full resolution N/A initial identification N/A other pathology
Swelling	Yes / No	Count new focal lesions 1. By region and side 2. By largest cross-sectional diameter Score 0-0 Score 1- Monofocal ≤ 2 cm Score 2- Multifocal ≤ 2 cm Score 3- Any lesion > 2 but < 4 cm Score 4- Any lesion >4 cm Score 5- Entire lobe	Increase ARIA-E ARIA-E unchanged Partial resolution Full resolution N/A initial identification N/A other pathology

### Statistical analysis

The sensitivity and specificity of ARIA-E detection with NAT alone and with SUB were measured. In this study, the gold standard true-positives were the cases determined to have ARIA-E lesions by consensus after conducting the inter-rater reliability study [7] before using SUB. This is a conservative approach since some false-negatives based on subtraction may be real ARIA-E. We evaluated the number of ARIA-E cases in which ≥1 neuroradiologist(s) rated a score of ≥1 in at least one brain region. Moreover, a majority vote, i.e. the number of cases in which at least three of the five raters rated a score of ≥1 in one or more brain regions, was assessed.

Observer variation was quantified in absolute terms through agreement and in relative terms through reliability [27]. The interobserver reliability, i.e. the consistency among the scores of the five raters, was assessed by determining the intraclass correlation coefficient (ICC). This was calculated as the ratio between subject variability and total variability, and a two-way mixed model measuring the absolute agreement was chosen because of the skewed scores distribution in a fixed ordinal scale [28]. The ICC was compared between the NAT and SUB group for all ARIA-E features. The ICC among all raters was measured in all 75 patients for each ARIA-E finding in all the six regions and both hemispheres. Concordance was considered poor-to fair with ICCs ≤0.40; moderate with ICC 0.41–0.60; good with ICC 0.61–0.80; and excellent with ICC ≥0.80 [29, 30]. The diagnostic accuracy of NAT and SUB was reported in terms of sensitivity and specificity with 95 % confidence intervals (CIs).

The inter-rater agreement, i.e. interobserver variation among the five raters was assessed by taking into account the overall number of ARIA-E lesions in all brain areas and measuring the proportion of specific agreement [27]. Every score (range 0–5) of each rater was compared to all the other raters’ scores per subtype in each hemisphere (L/R), resulting in ten ratings combinations within each hemisphere, which were afterwards summed up. The specific agreement shows the concordance among neuroradiologists with respect to the presence of positive (presence of ARIA-E) and negative (absence of ARIA-E) ratings. We also tested the effect on agreement measures of increasing the ARIA-E positivity threshold to a score ≥2 points. Statistical analyses were conducted with the IBM SPSS for Windows, Version 22.0 (IBM Corp., Armonk, NY, USA).

## Results

**Fig. 2:**
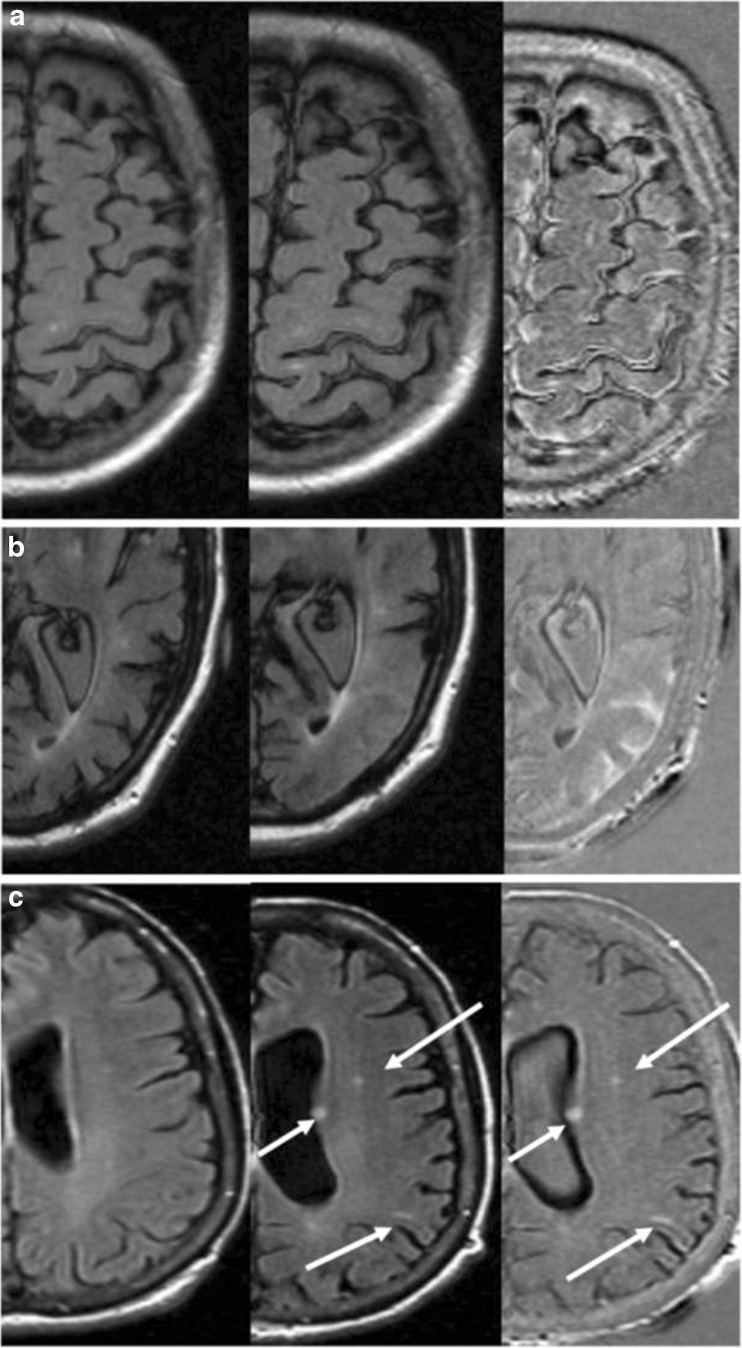
Details of three cases, with axial baseline FLAIR scan (left), the follow-up FLAIR scan (center) showing doubtful amyloid-related image abnormalities with vasogenic oedema and/or sulcal effusion (ARIA-E) findings and subtraction images (right) aiding in their detection (**a**), evaluation of their extent (**b**), and differential diagnosis (**c**). (**a**) Image artifacts prevent ARIA-E detection in FLAIR but the abnormalities are more visible on subtraction images. (**b**) The extension of the gyral swelling in the left occipital lobe is cumbersome to evaluate on FLAIR images but definitely more clear-cut in the subtraction images. (**c**) Small signal hyperintensities are visible on both FLAIR and subtraction images, hence their vascular origin may be excluded

**Fig. 3:**
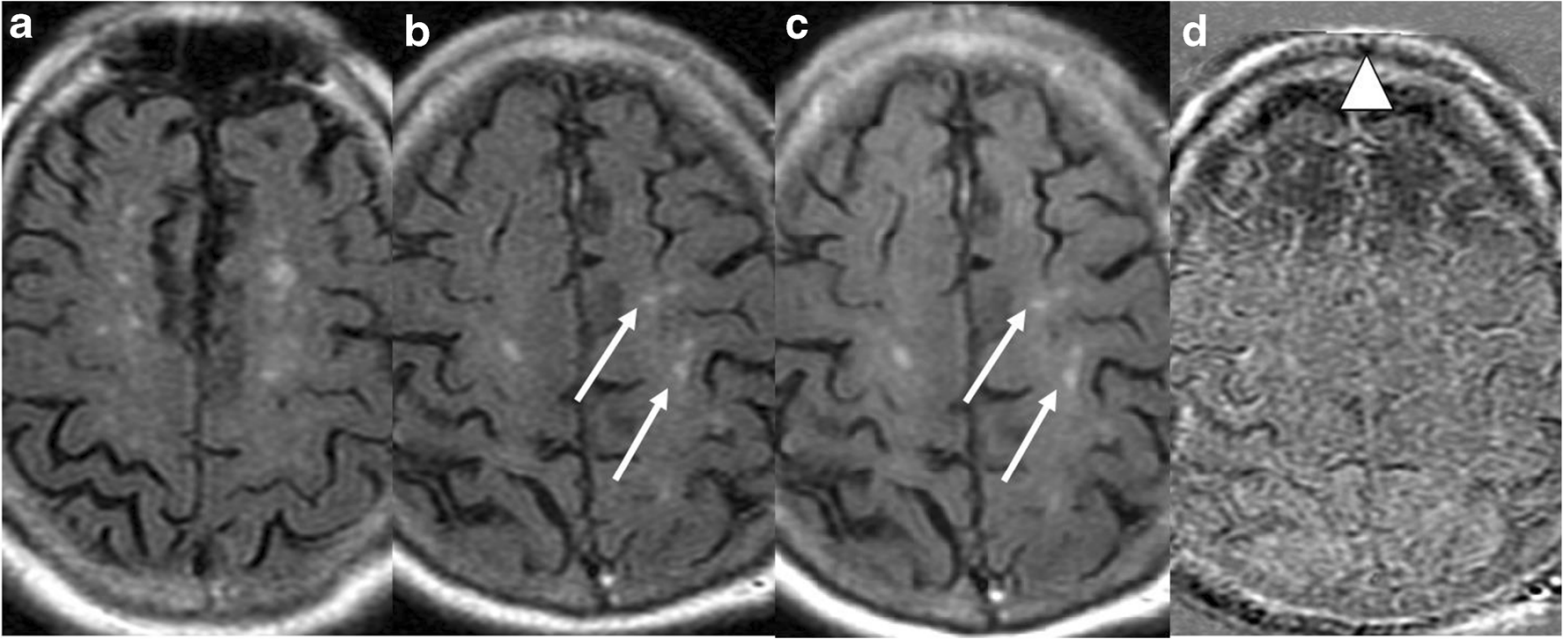
Some doubtful amyloid-related image abnormalities with vasogenic oedema and/or sulcal effusion (ARIA-E) lesions are detected on the left and right parietal areas (white arrows) they could not be confirmed/excluded on the subtraction image (**d**) due to angulation differences of the baseline scan (**a**) and the follow-up scan (**b**). Although the registered baseline (**c**) is comparable with the follow-scan, a typical misregistration artifact occurred (white triangle). Two out of five neuroradiologists rated this case as a parenchymal hyperintensity ARIA-E lesion

In 16 out of 75 patients only the TE was slightly different between the baseline and follow-up scans with a mean difference of 7.24 ms. The use of subtraction led to an increased number of abnormal cases and areas (readings), although there were more ‘false-positive’ cases in the SUB. This increased detection of possible ARIA-E cases and suspected areas using SUB might by caused either by showing additional cases or additional lesions in positive cases. SUB were especially sensitive to detect swelling (Figs. 1 and 2). The increased sensitivity led to detection of additional small hyperintensities on SUB, which were marked as ARIA-E, even though some raters reported doubts regarding their vascular origin (Fig. 2). The neuroradiologists reported insufficient quality of 19 SUB, including five of the 29 cases with ARIA-E. On the other hand, 16 SUB, 11 of which were ARIA-E positive, were marked as highly beneficial for the rating sake. Although some discrepant readings were reported (Fig. 3), neuroradiologists indicated that SUB were helpful in the detection or exclusion of ARIA-E abnormalities when image quality was sufficient.

**Fig. 4:**
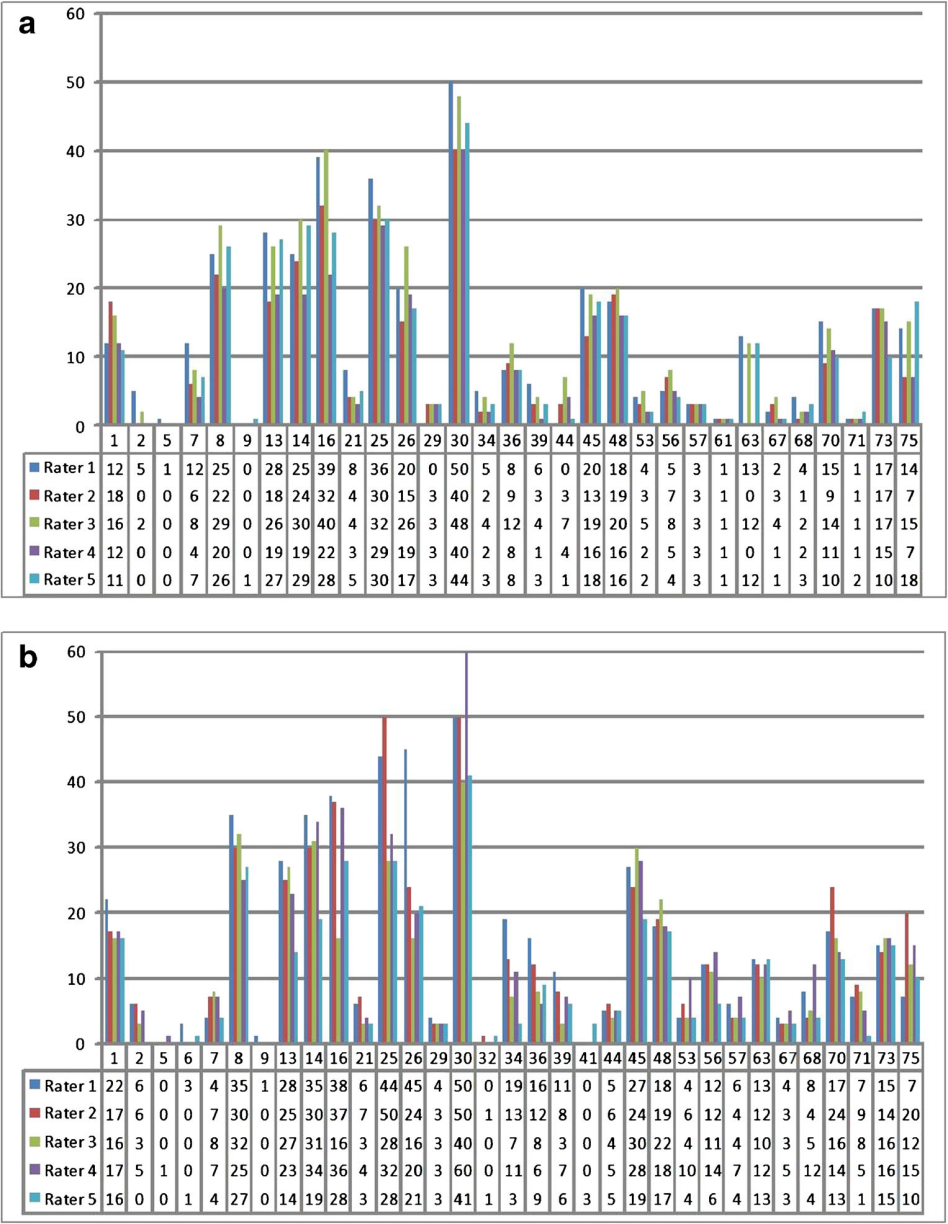
Sum of the scores per rater in all cases that were rated positively by a minimum of one rater. The vertical axis shows the sum of the highest scores in all amyloid-related image abnormalities with vasogenic oedema and/or sulcal effusion (ARIA-E) subtypes of all 12 brain areas. (**a**) In the native image group the horizontal axis shows 31 patients, including 29 ARIA-E cases and two false positives (FPs) (cases 5 and 9). (**b**) In the subtraction group 33 cases are shown, including 28 ARIA-E, one false negative FN (case 61) and five FPs (cases 5, 6, 9, 32 and 41)

The sensitivity and specificity of NAT and SUB evaluations, based on single-rater scores, are reported in Table 3A and B. Assessing the ARIA-E positive cases using only NAT, the detection of ARIA-E resulted in no missed ARIA-E cases (false-negatives, FN) and two false-positives (FP). Using SUB, one FN case and five FP cases were found (Fig. 4). In total there were 29 ARIA-E cases and 46 non-ARIA-E cases scored by five raters, resulting in 375 readings. The detection of ARIA-E in all readings is shown in Table 3C and D. Using NAT, one rater found six FN and two FP reading (score 1). Using SUB, five FN (all five raters missed one case) and seven FP readings were reported (three cases with one positive reading, two cases with two positive readings).

**Table 3:**
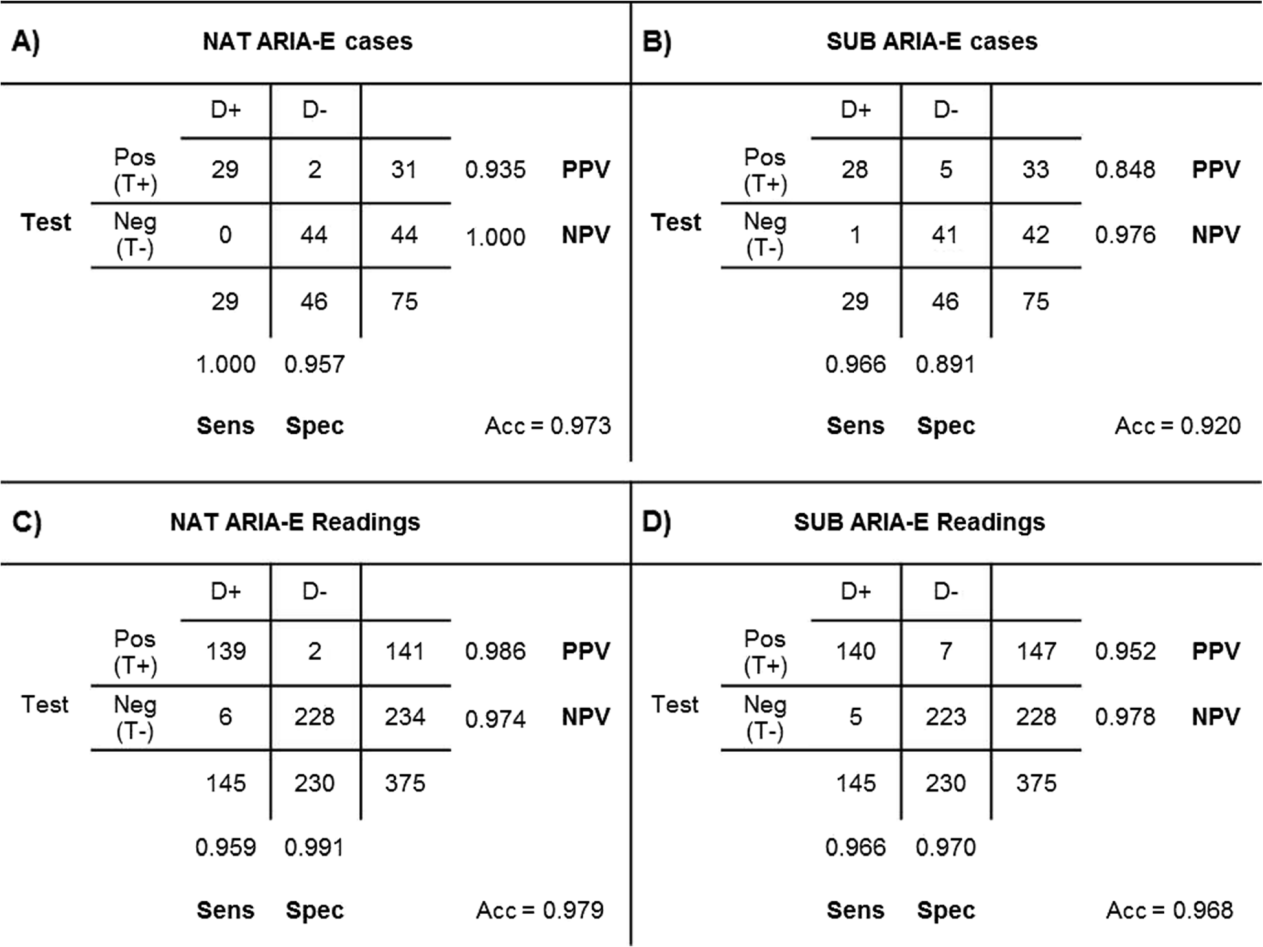
The sensitivity (Sens), specificity (Spec), positive predictive value (PPV) and negative predictive value (NPV) based on a positive/negative test (T+/T-) in ARIA cases (D+) and non-ARIA cases (D-) in (**A**) native images (NAT) and (**B**) subtraction images (SUB) if ≥1 rater scored a case with 1 or higher. In sections **C** and **D** the detection is shown in all readings of five raters in all 75 case

When assessing the ARIA-E detection by majority vote (i.e. a minimum of three raters gave a score of at least 1 in one brain region), no FP or FN cases occurred in the NAT and SUB group, resulting in 100 % sensitivity and specificity. The highest rating among all the ARIA-E characteristics per region was selected and summed up, and the results for each neuroradiologist with and without the use of SUB are reported in Fig. 4a and b, respectively. The range of scores was wider in most cases in the SUB compared to NAT.

The ICC scores with 95 % CIs are reported in Table 4. Overall, the ICC scores of the SUB tended to be lower compared to NAT, even though no statistically significant difference was found. Excellent inter-rater agreement was measured with the NAT and the SUB for sulcal hyperintensity, highest score of the subtypes, and sum of sulcal hyperintensity and gyral swelling.

**Table 4:** Intra-class correlation coefficient of the five raters of the ARIA-E imaging features in all 75 patients and in the ARIA-E cases only, averaged by all regions in each hemisphere. As is shown, the ICC of the subtraction group is slightly lower than the ICC of the native image group. The ICC score in the subtraction group of PH, SH and SW ranged from moderate to good (0.6–0.8). The combination of sulcal hyperintensity and gyral swelling resulted in an excellent agreement (a score above 0.8)

	MRI	ICC		ICC	
ARIA-E imaging features		n=75	95 % CI	n=29	95 % CI
Parenchymal hyperintensity	Native	0.630	0.60-0.66	0.611	0.56-0.56
	Subtraction	0.592	0.56-0.62	0.580	0.52-0.65
Sulcal hyperintensity	Native	0.800	0.78-0.82	0.780	0.75-0.81
	Subtraction	0.745	0.72-0.77	0.721	0.66-0.77
Swelling	Native	0.683	0.66-0.71	0.634	0.58-0.69
	Subtraction	0.606	0.58-0.64	0.576	0.51-0.65
Highest score of subtypes	Native	0.912	0.89-0.93	0.810	0.73-0.87
	Subtraction	0.823	0.81-0.84	0.824	0.79-0.86
Sulcal hyperintensity and gyral swelling	Native	0.836	0.82-0.85	0.811	0.78-0.84
	Subtraction	0.815	0.80-0.83	0.805	0.76-0.84

The ICC of sulcal hyperintensity was excellent in the NAT and good in the SUB. The ICC of the highest score of the three subtypes was excellent for both modalities. The ICC of the sum score of sulcal hyperintensity and sulcal swelling was excellent in the NAT and good in the SUB.

The proportion of specific agreement of all subtypes is shown in Table 5. The overall agreement was excellent for both modalities in all three ARIA subtypes (range 88.8–95.5 %). The positive agreement for sulcal hyperintensity was good; for swelling it was good in the NAT and moderate in the SUB; and for parenchymal hyperintensity was moderate in both groups. The negative agreement was excellent for all subtypes. Setting the cut-off level to ≥2 for a positive test result, the overall agreement remained consistent (0.5–1.2 % increase), while the positive agreement decreased in parenchymal and sulcal hyperintensity as well as in the swelling subtype.

**Table 5:** Specific agreement amyloid-related image abnormalities with vasogenic oedema and/or sulcal effusion (ARIA-E) characteristics and combination of sulcal hyperintensity and gyral swelling

Parietal hyperintensity		Native images			Subtraction images		
		Total	n	Estimate % (CI)	Total	n	Estimate % (CI)
Overall agreement	Agreement	9,000	8,591	95.5 (95.0–95.9)	9,000	8,595	95.5 (95.1–96.0)
	Agreement+1	9,000	8,775	97.5 (97.2–97.8)	9,000	8,812	97.9 (97.6–98.2)
Cut-off 0 vs. ≥1	Mean agreement	9,000	8,680	96.4 (96.0–96.8)	9,000	8,694	96.6 (96.2–97.0)
	Positive agreement	376	216	57.4 (52.3–62.5)	332	179	53.9 (48.4–59.4)
	Negative agreement	8,624	8,464	98.4 (97.8–98.4)	8,668	8,515	98.2 (97.9–98.5)
Cut-off 0–1 vs. ≥2	Mean agreement	9,000	8,764	97.4 (97.0–97.7)	9,000	8,798	97.8 (97.4–98.1)
	Positive agreement	262	144	55.0 (48.7–61.1)	218	117	53.7 (46.8–60.4)
	Negative agreement	8,738	8,620	98.6 (98.4–98.9)	8,782	8,681	98.9 (98.6–99.1)
Sulcal hyperintensity		Native images			Subtraction images		
		Total	n	Estimate % (CI)	Total	N	Estimate % (CI)
Overall agreement	Agreement	9,000	8,362	92.9(92.4–93.4)	9,000	8,105	90.1 (89.4–90.7)
	Agreement+1	9,000	8,693	96.6 (96.2–97.0)	9,000	8,503	94.5 (94.0–94.9)
Cut-off 0 vs. ≥1	Mean agreement	9,000	8,678	96.4 (96.0–96.8)	9,000	8,500	94.4 (94.0–94.9)
	Positive agreement	758	597	78.8 (75.7–81.6)	932	682	73.2 (70.2–76.0)
	Negative agreement	8,242	8,081	98.0 (97.7–98.3)	8,068	7,818	96.9 (96.5–97.3)
Cut-off 0–1 vs. ≥2	Mean agreement	9,000	8,718	96.9 (96.5–97.2)	9,000	8,568	95.2 (94.7–95.6)
	Positive agreement	644	503	78.1 (74.7–81.2)	820	604	73.7 (70.5–76.6)
	Negative agreement	8,356	8,215	98.3 (98.0–98.6)	8,180	7,964	97.4 (97.0–97.7)
Gyral swelling		Native images			Subtraction images		
		Total	n	Estimate % (CI)	Total	n	Estimate % (CI)
Overall agreement	Agreement	9,000	8,247	91.6 (91.0–92.2)	9,000	7,989	88.8 (88.1–89.4)
	Agreement+1	9,000	8,532	94.7 (94.2–95.2)	9,000	8,338	92.6 (92.1–93.2)
Cut-off 0 vs. ≥1	Mean agreement	9,000	8,502	94.5 (94.0–94.9)	9,000	8,295	92.2 (91.6–92.7)
	Positive agreement	788	539	68.4 (65.0–71.6)	902	549	60.9 (57.6–64.1)
	Negative agreement	8,212	7,963	97.0 (96.6–97.3)	8,099	7,746	95.6 (95.2–96.1)
Cut-off 0–1 vs. ≥2	Mean agreement	9,000	8,550	95.0 (94.5–95.4)	9,000	8,374	93.0 (92.5–93.6)
	Positive agreement	718	493	68.7 (65.1–72.0)	808	495	61.3 (57.8–64.6)
	Negative agreement	8,282	8,057	97.3 (96.9–97.6)	8,192	7,879	96.2 (95.7–96.6)
Combination sulcal hyperintensity and gyral swelling		Native images			Subtraction images		
		Total	n	Estimate % (CI)	Total	N	Estimate % (CI)
Overall agreement	Agreement	9,000	8,279	92.0 (91.4–92.5)	9,000	8,027	89.2 (88.5–89.5)
	Agreement+1	9,000	8,668	96.3 (95.9–96.7)	9,000	8,547	95.0 (94.5–95.4)
Cut-off 0 vs. ≥1	Mean agreement	9,000	8,670	96.3 (95.9–96.7)	9,000	8,550	95.5 (94.2–95.4)
	Positive agreement	968	803	83.0 (80.4–85.3)	1,158	933	80.6 (78.2–82.8)
	Negative agreement	8,032	7,867	98.0 (97.6–98.2)	7,842	7,617	97.1 (96.7–97.5)
Cut-off 0–1 vs. ≥2	Mean agreement	9,000	8,690	96.6 (96.2–96.9)	9,000	8,608	96.5 (96.2–96.7)
	Positive agreement	844	689	81.6 (78.9–84.2)	1,628	721	69.5 (66.6–72.3)
	Negative agreement	8,156	8,001	98.1 (97.8–98.4)	7,372	7,762	97.5 (97.2–97.9)

*n= Sum of points where raters had an agreement on

**Agreement + 1 = Overall agreement of the raters including positive and negative agreement. For theseis results a cut-off ≥>=2 was chosen, therefore scores of 0 and 1 were considered as an absence of an ARIA-E

No relevant variations in positive and negative agreement were observed when combining the abnormalities subtypes (range -0.3 % to +1.4 %). In contrast, when sulcal hyperintensity and sulcal swelling were combined, the overall agreement increased to excellent in both groups. Finally, increasing the positivity cut-off to ≥2 raters scoring ARIA-E, the positive agreement increased to excellent.

## Discussion

Amyloid-related imaging abnormalities occur in AD patients undergoing immune therapy. In this study, we evaluated the sensitivity, specificity, inter-rater reliability and specific positive and negative agreement among five experienced neuroradiologists detecting and classifying ARIA-E with and without the use of SUB. The raters used the subtraction technique in addition to standard axial FLAIR images to identify and rate ARIA-E. Discrepancies in ratings occurred mostly in the presence of sulcal hyperintensities or when differentiating small parenchymal hyperintensities from vascular lesions.

The detection of ARIA-E was high in the NAT and SUB, but the specificity was lower using the SUB. Our results with the use of NAT only were in line with previous studies [15]. We registered more FP cases with SUB compared to NAT (13 and two FP, respectively). The sensitivity and specificity with NAT and SUB by majority vote was excellent. Once an ARIA-E finding was detected, the neuroradiologists tried to rate it as either parenchymal hyperintensity, sulcal hyperintensity or sulcal swelling, but this categorisation was challenging especially when distinguishing between sulcal hyperintensity and sulcal swelling. The ICCs of sulcal hyperintensities was excellent using the NAT (0.915) and good (0.740) using SUB. Lower ICC scores were reported for sulcal swelling in NAT and SUB (0.660 and 0.440, respectively), due to inconsistencies in raters’ interpretation of the characteristics. Because of the lack of signal hyperintensities, the identification of the boundaries of swelling was challenging on the NAT and likely caused variations among raters’ scores. ARIA-E rating for gyral swelling and parenchymal hyperintensities improved when using only NAT, but, when combining the two subtypes together, the ICC increased to excellent in both NAT and SUB groups. The combination of sulcal hyperintensity and gyral swelling in the ARIA-E rating scale would therefore provide higher inter-rater reliability.

When assessing all the brain areas together for each of the 75 AD patients, the inter-rater reliability among all neuroradiologists ranged between good and excellent, except for swelling, for which moderate agreement was reported. Since the total of the scores in each of the 12 regions per subtype approached a nominal scale, the ICC statistical test was employed to describe the scores’ variation for each abnormality subtype [30]. All brain regions showing no ARIA-E abnormalities were also taken into account, which led to reduced CIs. Among the 29 ARIA-E positive patients, the CIs of the ICCs were wider compared to the whole set of 75 patients. Thus, in the clinical setting, summing the scores of all abnormalities in each brain area may result in a low ICC and hence in an imprecise lesion load estimate.

The specific agreement on each ARIA-E subtype demonstrated no statistically significant differences with or without the use of SUB. The positive agreement was slightly lower in the subtraction group, but the performance of this test increased with respect to the ICC when summing all the brain areas. The total number of lesions rated as parenchymal hyperintensities was three times lower than the number of lesions rated as sulcal hyperintensities or gyral swelling. These two latter subtypes also showed higher positive agreement, probably thanks to extra information provided by the SUB. On the other hand, their use also increased the ambiguity in choosing between them, even though the total amount of ARIA-E lesions detected remained unchanged.

When the cut-off level for positive agreement was set to a score ≥1, a greater number of ARIA-E parenchymal hyperintensities was detected with NAT instead of with the SUB. No statistically significant changes were reported for overall agreement with a cut-off level ≥2 for a positive test. Placing a higher cut-off was an attempt to filter out small ambiguous lesions due to artefacts. Nevertheless, this did not result in an alteration in the value of positive agreement. Combining sulcal hyperintensity and swelling subtypes, the positive agreement increased from good to excellent in both the NAT and SUB groups, since categorisation was not necessary anymore and hence a more uniform rating was obtained.

We think that the quality of the FLAIR images was a major factor influencing the raters’ agreement in both the native and subtraction groups. A high rate of FP was detected when using SUB due to large slice thickness resulting in subtraction artefacts, which had some aspect similar to an ARIA-E lesion. This similarity caused difficulties distinguishing between artefacts and a possible ARIA-E findings. While it was expected that the inter-rater reliability would have been slightly lower with SUB as compared to NAT because of their methodological similarity, it could have been possible that the FP detected with SUB were actually ARIA-E lesions missed by the gold standard read. However not all raters agreed on this point.

The acquisition protocols differed among the acquisition centres, resulting in differences between patients in TR/TE, flip-angle, voxel size, acquisition matrix, field of view and image contrast. This could have led to differences in the incidence or contrast of (pulsation) artefacts and, in some cases, suitability for registration, since the same global scaling and registration algorithms were used in all cases. The use of isotropic 3D-FLAIR is likely to improve not only the detection and characterisation of cortical lesions, but also SUB performance, as shown for multiple sclerosis [31–33] thanks to higher spatial resolution signal-to-noise ratio compared with 2D multislice acquisition, and decreased pulsation artefacts [34].

Even though the acquisition protocol was identical within patients, variations among centres might have deleterious effects on accuracy in registration. In addition, slice repositioning differences could have caused suboptimal co-registration. Moreover, misregistration could be due to non-linear deformations (e.g. swelling side-effects of the immunisation treatment) for which an elastic deformation (FNIRT) algorithm could be considered instead of FLIRT. However, FNIRT would countervail important ARIA-E findings’ characteristics and nullify valuable aspects of swelling or sulcal hyperintensity. Furthermore, most of the suboptimal SUB were reported in non-ARIA cases and showed linear artefacts in a pattern of black and white lines.

Inter-rater reliability and agreement for ARIA-E monitoring may be improved through radiologists’ training or through a semi-quantitative rating scale such as the one used in this study, including all ARIA-E subtypes. A severity scale for each brain region, would allow for monitoring on a higher level.

## Conclusion

Subtraction MRI has potential as a visual aid increasing the sensitivity of ARIA-E assessment. However, in order to improve its usefulness isotropic acquisition and enhanced training are required. The ARIA-E rating scale may benefit from combining sulcal hyperintensity and swelling.

## Abbreviations

AD: Alzheimer’s disease
ARIA: Amyloid-related image abnormalities
ARIA-E: Amyloid-related image abnormalities with vasogenic oedema and/or sulcal effusion
ARIA-H: Amyloid-related image abnormalities with hemosiderin deposits and microbleeds
Aß: Amyloid-ß
CSF: Cerebrospinal fluid
FLAIR: Fluid-attenuated inversion recovery
FLIRT: FMRIB’s linear image registration tool
ICC: Intraclass correlation coefficient
IRR: Inter-rater reliability
MMSE: Mini-Mental-State-Examination
MRI: Magnetic resonance imaging
NAT: Native FLAIR images (baseline and follow-up)
PH: Parenchymal hyperintensity
SH: Sulcal hyperintensity
SUB: Subtraction images
SW: Gyral swelling
